# Robust Walking for Humanoid Robot Based on Divergent Component of Motion

**DOI:** 10.3390/mi13071095

**Published:** 2022-07-11

**Authors:** Zhao Zhang, Lei Zhang, Shan Xin, Ning Xiao, Xiaoyan Wen

**Affiliations:** College of Electrical and Information Engineering, Beijing University of Civil Engineering and Architecture, Beijing 102612, China; bjjzdxzz@163.com (Z.Z.); xinshan@bucea.edu.cn (S.X.); xiaoning@bucea.edu.cn (N.X.); wenxiaoyan@bucea.edu.cn (X.W.)

**Keywords:** humanoid robot, model predictive control, divergent component of motion, gait planning, extended Kalman filter

## Abstract

In order to perform various complex tasks in place of humans, humanoid robots should walk robustly in the presence of interference. In the paper, an improved model predictive control (MPC) method based on the divergent components of motion (DCM) is proposed. Firstly, the humanoid robot model is simplified to a finite-sized foot-pendulum model. Then, the gait of the humanoid robot in the single-support phase (SSP) and double-support phase (DSP) is planned based on DCM. The center of mass (CoM) of the robot will converge to the DCM, which simplifies the feedback control process. Finally, an MPC controller incorporating an extended Kalman filter (EKF) is proposed to realize the tracking of the desired DCM trajectory. By adjusting the step duration, the controller can compensate for CoM trajectory errors caused by disturbances. Simulation results show that—compared with the traditional method—the method we propose achieves improvements in both disturbed walking and uneven-terrain walking.

## 1. Introduction

In recent years, research on humanoid robot has received high priority. As the first step for humanoid robots to replace humans, it is necessary to ensure robust walking for humanoid robots, especially when walking on uneven terrain [1]. The research on bipedal robust walking of humanoid robots has become one of the most challenging research topics [2].

When walking on uneven ground, humanoid robots need the ability to adjust their gait to avoid falling caused by disturbance. Due to the high dimension of contact force between the sole of the humanoid robot and the ground, the robot’s leg-motion control is very difficult [3,4]. Nowadays, there are many theories for the balance control of humanoid robots, such as the zero-moment point (ZMP) [5] and DCM [6] criteria of stability, etc. In addition, an excellent controller is necessary to ensure robust walking of robots. Since MPC controllers can solve optimization problems with multiple constraints, they are widely used in the field of bipedal robot walking.

In this paper, an improved MPC controller is proposed to track the desired DCM trajectory, which enhances robustness of the robot suffering from disturbance. Desired DCM trajectories for the SSP and the DSP are generated. An inverted pendulum model with a finite-sized foot [7] is used in gait planning. It takes into account the ankle-joint moment to overcome the low robot dexterity caused by insufficient ankle drive. In addition, the MPC controller expanded by an EFK is designed, which can compensate for a certain range of disturbances by adjusting step duration during walking. The simulation results show that the hybrid controller we propose can change step length and width by adjusting step duration.

The rest of the paper is structured as follows. Related works on robot bipedal motion are introduced in Section 2. Section 3 shows the simplified kinetic model and gait planning. In Section 4, the MPC controller expanded by an EKF is designed. Simulation results and discussions are described in Section 5, while Section 6 concludes the paper.

## 2. Related Works

A lot of concepts have been proposed for the stability control of bipedal walking. For example, the foot-rotation indicator (FRI) point was defined in [8] as a point on the foot/ground-contact surface where the net ground-reaction force would have to act to keep the foot stationary. To ensure the robot has no foot rotation, the FRI point must remain within the convex hull of the foot-support area [9,10]. However, the FRI point is difficult to calculate. Based on FPI, Ono et al. [11] presented a permissible amount of falling risk evaluation that was calculated by plantar contact points of the robot, acceleration value of center of gravity (CoG), and reaction force. This method can assess the risk of falling when the robot is walking, while ZMP, which was defined as the center point of the reaction forces, is the first indicator to measure the stability margin of bipedal walking [12,13]. Usually, the ZMP criterion is combined with the linear inverted pendulum model (LIPM) to achieve robust walking of the bipedal robot [14,15]. Kajita et al. [16] presented 3D-LIPM to analyze 3D walking control of a bipedal robot in which motion is constrained to an arbitrarily defined plane. Based on the geometric properties of 3D-LIPM, they planned the robot’s walking trajectory and applied it to a simulated robot. Furthermore, Kajita et al. [17] designed a linear inverted pendulum trajectory tracking controller based on CoM/ZMP. The main contribution of them was the application of body posture. They also used HRP-4C to evaluate the method. Unlike the approach proposed by Kajita, Wieber [18] designed an MPC controller to track the desired ZMP trajectory. He expressed the control problem as a QP problem, thus dealing with the robot system with nonlinear constraints. To avoid robots falling, Yu et al. [19] proposed a new landing-point adjustment algorithm. They measured changes in the acceleration of the robot’s CoM and mapped changes in ZMP onto the new support area based on variations. However, the conventional LIPM has strict limits on the height and angular momentum of the CoM, which greatly limit further robot application [20,21,22]. To solve this problem, scholars have improved the LIPM by considering the changes in height and angular momentum of the CoM. It requires more control variables and optimization constraints, which make ZMP difficult to calculate and control. Heerden [23] presented a trajectory generator for humanoid robots that can simultaneously consider various constraints, including nonlinear ZMP constraints. He transformed the control problem into a quadratically constrained quadratic program problem. However, this method needs better hardware and longer computing time. Caron et al. [24] analyzed the capturability of a variable-height linear inverted pendulum (VHIP) when walking on uneven terrain. They used the VHIP model to plan gait, and they gave a complicated method to optimize the capture problem.

In addition to ZMP, the DCM algorithm plays an extremely important role in the balance theory of humanoid robots [25,26,27]. Pratt et al. [28] proposed the concept of capture points (2D-DCM) to clarify when and where the robot should take steps to avoid falling, and they used capture points to implement the push recovery of bipedal robots. The capture point is defined as a point on the ground where the robot can step in order to bring itself to a complete stop. Hof [29] proposed the concept of extrapolating the center of mass, which is equivalent to DCM. They used capture points to implement the push recovery of bipedal robots. Takenaka et al. [30] analyzed the state-space equations of LIPM and obtained the unstable component of the state space, then introduced the unstable component as a new concept—the DCM—which is equivalent to the capture point in 2D. They proved that planning gait and designing a controller based on DCM can increase the stability margin and robustness of a bipedal robot while walking. Since only the divergent component is considered, this method greatly reduces the computational complexity of predictive control and simplifies gait generation. Since then, in the case of large disturbances, experts in humanoid robotics have started using the DCM criterion for gait planning and controller design.

Englsberger et al. [31] proposed two control loop design methods, CPS and CPT, based on the 2D-DCM stability criterion. CPS was a real-time control method, which had high requirements on the hardware of the robot. CPT needed to generate the gait offline. A trajectory-tracking controller was designed in the paper and took the error between the desired and actual DCM as input. Then, Englsberger et al. [32] proposed a trajectory-tracking controller based on ZMP, which compensated for changes in CoM height and angular momentum during the walking process. Sedye et al. [33] analyzed the influence of the angular momentum generated by the swinging legs during high-speed walking, which predicted the angular momentum of CoM in the planning stage. The generator produced smooth and continuous trajectories based on the instantaneous DCM.

On the one hand, the traditional controller based on ZMP has difficulty dealing with complex nonlinear constraints caused by the change in CoM height [34]. On the other hand, to improve stability, many constraints, such as structural size and stability margin, must be considered [35]. MPC can solve nonlinear multiple constraint optimization problems, so it is widely used in humanoids [36,37,38]. Krause et al. [39] were the first to design an MPC controller based on the DCM. They used double closed-loop control, which takes ZMP as input, the outer is an MPC controller, and the inner is a ZMP controller. Griffin et al. [40] analyzed the variation in DCM during walking and presented a time-varying DCM. To generate a robust gait, an MPC frame was formulated based on time-varying DCM that took step position and rotation into account. Simulation results verified the effectiveness of the method. Shafiee-Ashtiani et al. [41] designed a robust stepping MPC controller that could adjust step location and CoP. Experimental results showed that the robot could walk in various environments by taking time-varying DCM into account. To decrease the reaction time, Kasaei et al. [42] decoupled the control and replanning procedures. They formulated an offline optimal controller based on Linear–Quadratic–Gaussian (LQG) control [43] to achieve a reference trajectory-tracking process. Simulation in Matlab showed the capability of this algorithm. However, none of these methods consider the effect of noise when optimizing. We used EKF [44] to estimate the cost function to improve the robustness of bipedal walking.

## 3. Gait Planning for Humanoids

To adapt to real-world environments, bipedal robots should have human-like walking patterns [45]. The reproduction of a walking cycle for a walking robot is based on the generation of a step sequence [46]. Each step is composed of two successive phases: the single-support phase (SSP) and double-support phase (DSP) [47]. In the SSP, the foot of the swing leg does not directly affect any force, and the ZMP is in the support area formed by the support foot. In the DSP, both feet are subject to the reaction force of the ground at the same time. The support area at this time is a quadrilateral formed by two feet. In this phase, the ZMP moves from the support area formed by the left (right) foot to the support area formed by the right (left) foot [48]. The transfer of the ZMP between the feet allows the robot to keep moving forward. In addition, since the robot has a larger support area in the DSP, ZMP has a larger stability margin in the DSP [49]. Overall, in order to make the robot walk stably, the SSP and DSP are indispensable. Next, we introduce the inverted pendulum model used and the gait planning of the SSP and DSP.

### 3.1. LIPM with Finite-Sized Feet

**Figure 1:**
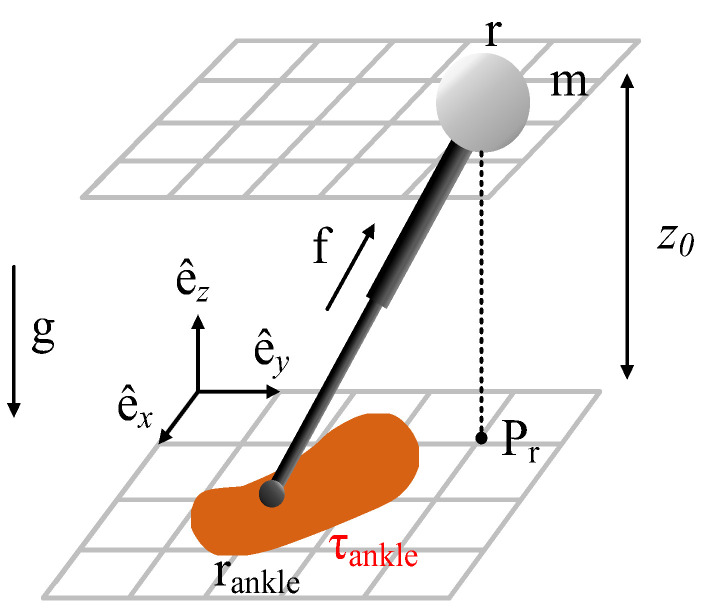
LIPM with finite-sized feet.

The simplified model of bipedal walking is shown in Figure 1. It takes the ankle torque into account, which makes the CoP controllable [50]. As shown in Figure 1, m represents the total mass of the robot. We assume that the mass of the robot is concentrated in one point $r={\left(\begin{array}{ccc} x & y & z \end{array}\right)}^{T}$, which moves on the same level and *z = z*_0_. $f={\left(\begin{array}{ccc} f_{x} & f_{y} & f_{z} \end{array}\right)}^{T}$ is the reaction force of the floor acting on the ankle joint. $r_{\mathrm{ankle}}={\left(\begin{array}{ccc} x_{\mathrm{ankle}} & y_{\mathrm{ankle}} & 0 \end{array}\right)}^{T}$ is the projection of the ankle joint onto the ground. $\tau_{\mathrm{ankle}}={\left(\begin{array}{ccc} \tau_{\mathrm{ankle},x} & \tau_{\mathrm{ankle},y} & \tau_{\mathrm{ankle},z} \end{array}\right)}^{T}$ is the ankle torque. Pr is the projection of the CoM position on the ground. and $g={\left(\begin{array}{ccc} 0 & 0 & -g \end{array}\right)}^{T}$ is the gravitational acceleration vector.

The force and moment equations of LIPM with finite-sized feet are as follows [51]:

(1) $$m\ddot{r}=f+mg$$

(2) $$-(r-r_{ankle})\times f{+\tau }_{ankle}=0$$

Due to the constraints of *z* = *z*_0_, $\dot{z}$ and $\ddot{z}$, both are zero, and it can be known from Formula (1) that $f_{z}=mg$. Taking it into Formula (2) to get *f_x_*, *f_y_* and $\tau_{\mathrm{ankle},z}$:

(3) $$\begin{array}{r} f_{x}{=m\omega }^{2}({x-x}_{\mathrm{ankle}})+\frac{\tau_{\mathrm{ankle},y}}{z_{0}}\\ f_{y}{=m\omega }^{2}({y-y}_{\mathrm{ankle}})+\frac{\tau_{\mathrm{ankle},x}}{z_{0}}\\ \tau_{\mathrm{ankle},z}=-\frac{\tau_{\mathrm{aankle},x}}{z_{0}}({x-x}_{\mathrm{ankle}})\\ -\frac{\tau_{\mathrm{ankle},y}}{z_{0}}({y-y}_{\mathrm{ankle}}) \end{array}$$

In summary, the dynamic equation of the bipedal walking model is:

(4) $$\ddot{r}=\omega^{2}(Pr-r_{\mathrm{cop}})$$

where $\omega =\sqrt{g/z_{0}}$.

$$\begin{array}{l} r_{\mathrm{cop}}=r_{\mathrm{ankle}}+\Delta r_{\mathrm{cop}}\\ \Delta r_{\mathrm{cop}}=-\frac{1}{\mathrm{mg}}\left(\begin{array}{c} \tau_{\mathrm{ankle},y}\\ -\tau_{\mathrm{ankle},x}\\ 0 \end{array}\right) \end{array}$$

Since the vertical motion of the CoM is fixed, Formula (4) can be written as Formula (5). (*x_c_*, *y_c_*) is the coordinate of COP.

(5) $$\left[\begin{array}{c} \dot{x}\\ \dot{y}\\ \ddot{x}\\ \ddot{y} \end{array}\right]=\left[\begin{array}{cccc} 0 & 0 & 1 & 0\\ 0 & 0 & 0 & 1\\ \omega^{2} & 0 & 0 & 0\\ 0 & \omega^{2} & 0 & 0 \end{array}\right]\left[\begin{array}{c} x\\ y\\ \dot{x}\\ \dot{y} \end{array}\right]+\left[\begin{array}{cc} 0 & 0\\ 0 & 0\\ -\omega^{2} & 0\\ 0 & -\omega^{2} \end{array}\right]\left[\begin{array}{c} x_{c}\\ y_{c} \end{array}\right]$$

After homogeneous transformation, the unstable component of the state-space formula, which is also called DCM, can be obtained:

(6) $$\xi =\left[\begin{array}{c} x_{D}\\ y_{D} \end{array}\right]=\left[\begin{array}{c} x+\dot{x}/\omega \\ y+\dot{y}/\omega \end{array}\right]$$

### 3.2. Single-Support Phase

**Figure 2:**
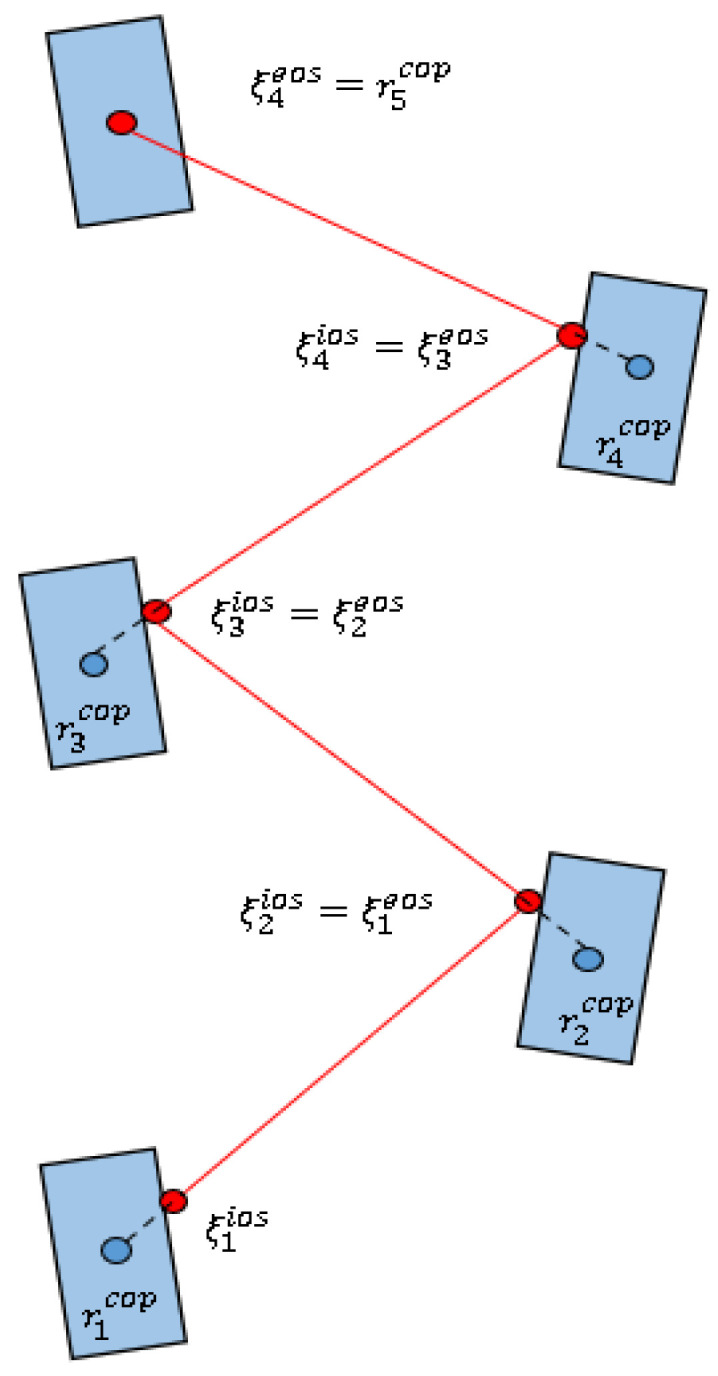
Desired DCM trajectory for the single-support phase.

The bipedal walking of the humanoid robot includes an SSP and a DSP. This paper assumes the transition of DCM and CoP between the feet is instantaneous when designing the SSP. It is shown in Figure 2.

Combining Formulas (4) and (6) leads to Formula (7):

(7) $$\dot{\xi }=\omega ({\xi -r}_{\mathrm{cop}})$$

The solution to Formula (7) is:

(8) $${\xi =r}_{\mathrm{cop}}+e^{\omega t}(\xi_{0}{-r}_{\mathrm{cop}})$$

where $\xi_{0}$ is the initial position of the DCM, time $t\in \left[0,t_{i}^{step}\right]$, and $t_{i}^{step}$ is the duration of step *i*.

In the last step, the DCM overlap with the CoP and the bipedal motion stops. The desired DCM position at the end of each step can be obtained by Formula (8):

(9) $$\{\begin{array}{c} \xi_{N-1}^{\mathrm{eos}}{=r}_{N}^{\mathrm{cop}}\\ \xi_{i-1}^{\mathrm{eos}}{=\xi }_{i}^{\mathrm{ios}}=r_{i}^{\mathrm{cop}}{+e}^{{-\omega t}_{i}^{\mathrm{step}}}(\xi_{i}^{\mathrm{eos}}{-r}_{i}^{\mathrm{eos}}) \end{array}$$

Combining Formulas (8) and (9), the desired trajectory of DCM can be obtained:

(10) $$\{\begin{array}{c} \xi_{i}(t){=r}_{i}^{\mathrm{cop}}{+e}^{\omega ({t-t}_{i}^{\mathrm{step}})}(\xi_{i}^{\mathrm{eos}}{-r}_{i}^{\mathrm{cop}})\\ {\dot{\xi }}_{i}(t){=\omega e}^{\omega ({t-t}_{i}^{\mathrm{step}})}(\xi_{i}^{\mathrm{eos}}{-r}_{i}^{\mathrm{cop}}) \end{array}$$

According to Formula (10), the expected DCM trajectory of each step can be obtained recursively.

### 3.3. Double-Support Phase

Considering only two SSPs in one walking cycle make the desired CoP trajectory discontinuous, the joint trajectory calculated by inverse kinematics is not continuous either, so, we plan the DSP trajectory. From Formula (7), we can get:

(11) $$r_{\mathrm{cop}}=\xi -\dot{\xi }/\omega$$

From Formula (11), it can be seen that the continuity of the desired CoP trajectory requires continuity of the first derivative of the DCM trajectory, We smooth the desired DCM trajectory using a cubic interpolation function [52]:

(12) $$\xi^{D}{=a}_{3}t^{3}+a_{2}t^{2}+a_{1}{t+a}_{0}$$

At the same time, Equation (12) must satisfy the boundary conditions of position and velocity:

$$\{\begin{array}{c} \xi_{i}^{\mathrm{inid}}{=r}_{i-1}^{\mathrm{cop}}{+e}^{\omega (t_{i-1}^{\mathrm{inid}}{-t}_{i-1}^{\mathrm{step}})}(\xi_{i-1}^{\mathrm{eos}}{-r}_{i-1}^{\mathrm{cop}})\\ {\dot{\xi }}_{i}^{\mathrm{inid}}{=\omega e}^{\omega (t_{i-1}^{\mathrm{inid}}{-t}_{i-1}^{\mathrm{step}})}(\xi_{i-1}^{\mathrm{eos}}{-r}_{i-1}^{\mathrm{cop}})\\ \xi_{i}^{\mathrm{eosd}}{=r}_{i}^{\mathrm{cop}}{+e}^{\omega (t_{i}^{\mathrm{eosd}}{-t}_{i}^{\mathrm{step}})}(\xi_{i}^{\mathrm{eos}}{-r}_{i}^{\mathrm{cop}})\\ {\dot{\xi }}_{i}^{\mathrm{eosd}}{=\omega e}^{\omega (t_{i}^{\mathrm{eosd}}{-t}_{i}^{\mathrm{step}})}(\xi_{i}^{\mathrm{eos}}{-r}_{i}^{\mathrm{cop}}) \end{array}$$

where, $\xi^{\mathrm{inid}}$, ${\dot{\xi }}^{\mathrm{inid}}$ are the initial position and velocity of the desired DCM trajectory in DSP, $\xi^{\mathrm{eosd}}$, ${\dot{\xi }}^{\mathrm{eosd}}$ are the end position and velocity of the desired DCM trajectory in DSP. $t^{\mathrm{inid}}$, $t^{\mathrm{eosd}}$ are the start and end time of the DSP.

The polynomial coefficients can be solved by Equation (13):

(13) $$\left[\begin{array}{c} a_{3_{i}}^{T}\\ a_{2_{i}}^{T}\\ a_{1_{i}}^{T}\\ a_{0_{i}}^{T} \end{array}\right]=\left[\begin{array}{cccc} 2/T_{i}^{3} & 1/T_{i}^{2} & -2/T_{i}^{3} & 1/T_{i}^{2}\\ -3/T_{i}^{2} & -2/T_{i} & 3/T_{i}^{2} & -1/T_{i}\\ 0 & 1 & 0 & 0\\ 1 & 0 & 0 & 0 \end{array}\right]\left[\begin{array}{c} {\left(\xi_{i}^{\mathrm{inid}}\right)}^{T}\\ {\left({\dot{\xi }}_{i}^{\mathrm{inid}}\right)}^{T}\\ {\left(\xi_{i}^{\mathrm{eosd}}\right)}^{T}\\ {\left({\dot{\xi }}_{i}^{\mathrm{eosd}}\right)}^{T} \end{array}\right]$$

where *T_i_* is the duration of the *i*-th DSP.

Thus, the DCM trajectory of the double-support stage is:

(14) $$\left[\begin{array}{c} \xi_{i}^{D}{\left(t\right)}^{T}\\ {\dot{\xi }}_{i}^{D}{\left(t\right)}^{T} \end{array}\right]=\left[\begin{array}{cccc} 3t^{2} & 2t & 1 & 0\\ t^{3} & t^{2} & t & 1 \end{array}\right]\left[\begin{array}{c} a_{3_{i}}^{T}\\ a_{2_{i}}^{T}\\ a_{1_{i}}^{T}\\ a_{0_{i}}^{T} \end{array}\right]$$

**Figure 3:**
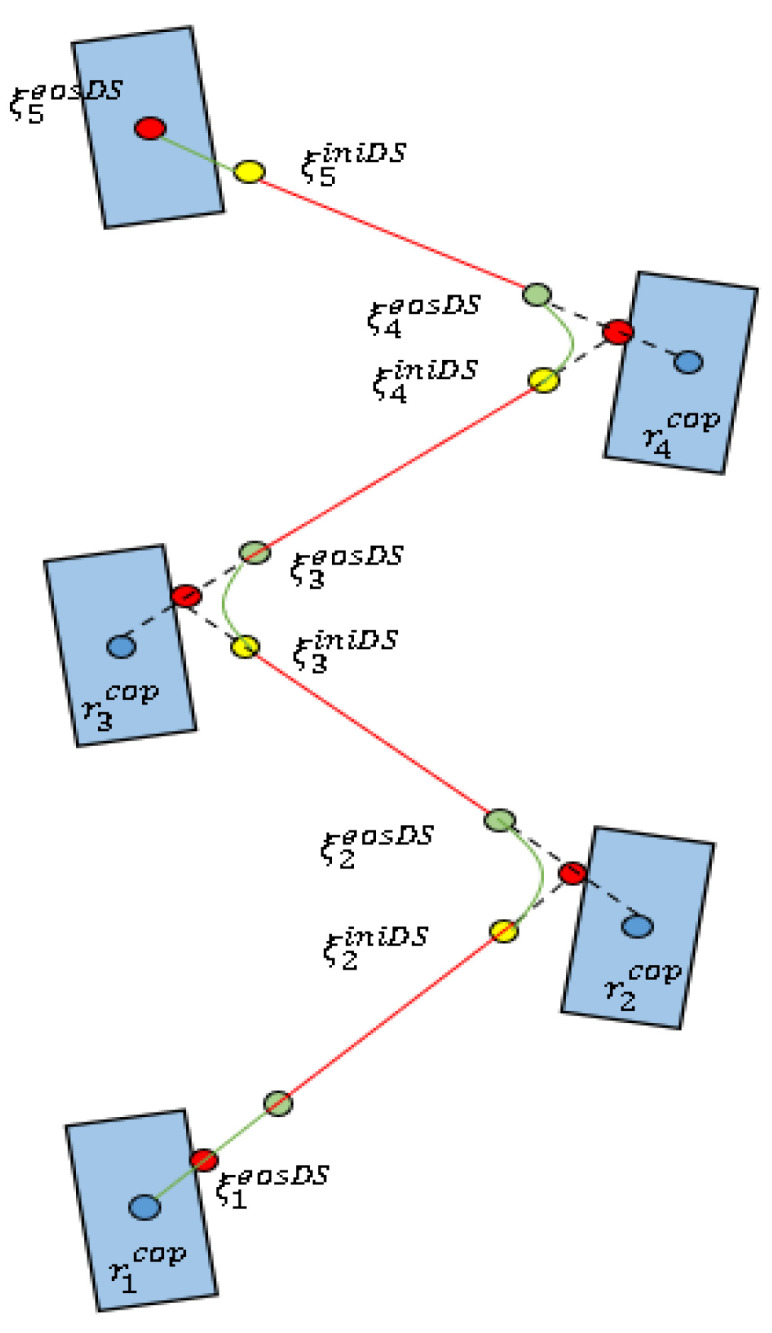
Desired DCM trajectory for the double-support phase.

The desired DCM trajectory is shown in Figure 3, the SSP trajectory is an exponential interpolation function, and the DSP is a cubic interpolation function.

After the above planning, the first derivative of the DCM trajectory is continuous everywhere and the CoP trajectory is continuous too.

## 4. Model Predictive Controller

Model predictive control is a control method that combines control and optimization [53,54]. It can solve optimization problems with multiple constraints. MPC relies on dynamic models of the process, most often linear empirical models obtained by system identification. The main advantage of MPC is the fact that it allows the current timeslot to be optimized, while keeping future timeslots in account [55].

### 4.1. Predictive Model

Given a continuous linear-time invariant system, such as Formula (7), it can be transformed as:

(15) $$\dot{\xi }={\omega \xi -\omega r}^{\mathrm{cop}}$$

However, the MPC model must be discrete. Thus, its discrete counterpart, evaluated using the zero-order hold (ZOH) technique, is:

(16) $$\xi_{k+1}=F_{d}\xi_{k}{+G}_{d}r_{k}^{\mathrm{cop}}$$

where *T* is the sampling time and *F_d_* and *G_d_* are:

(17) $$F_{d}{=e}^{\omega T},{\;G}_{d}=\int_{0}^{T}{-\omega e}^{-\omega \tau }d\tau$$

The prediction model of model predictive control can be obtained:

(18) $$\begin{array}{cc} \xi_{k+1} & ={F\xi }_{k}{+\mathrm{Gr}}_{k}^{\mathrm{cop}}\\ & =\left[\begin{array}{cc} e^{\omega T} & 0\\ 0 & e^{\omega T} \end{array}\right]\xi_{k}+\left[\begin{array}{cc} 1-e^{\omega T} & 0\\ 0 & 1-e^{\omega T} \end{array}\right]r_{k}^{\mathrm{cop}}\\ y_{k} & {=C}_{d}\xi_{k} \end{array}$$

So, the prediction model based on state-space variables is:

(19) $${\tilde{y}}_{k+1}{=P}_{x}{\tilde{\xi }}_{k}{+H}_{x}{\tilde{r}}_{k}^{\mathrm{cop}}$$

where *P_x_* is the matrix of coefficients formed by *F*. *H_x_* is the matrix of coefficients formed by *F* and *G*, and ${\tilde{y}}_{k+1}$, ${\tilde{\xi }}_{k}$, ${\tilde{r}}_{k}^{\mathrm{cop}}$ are deviations of output, variable, and control signal respectively.

### 4.2. Constraints of Model Predictive Control

**Figure 4:**
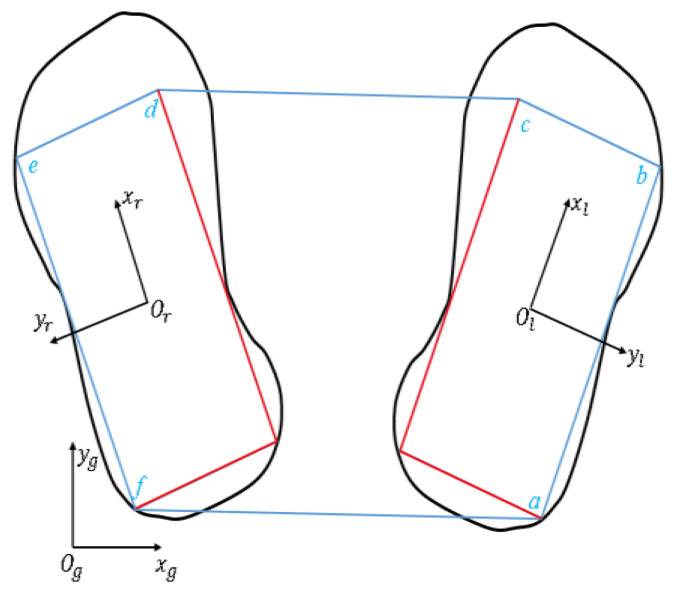
Support area of bipedal walking.

To ensure the viability of the gait, it is desired that the CoP cannot leave the support area created by the foot. The blue polygon in Figure 4 is the support polygon area of the DSP, and the red polygon is the support area of the SSP.

The equation of the straight line ab is:

(20) $$y=\frac{{x-b}_{x}}{a_{x}{-b}_{x}}a_{y}+\frac{{x-a}_{x}}{b_{x}{-a}_{x}}b_{y}$$

The next step (taking the left foot as an example) expects the CoP needs to be satisfied:

(21) $$p_{y}\geq \frac{p_{x}{-b}_{x}}{a_{x}{-b}_{x}}a_{y}+\frac{p_{x}{-a}_{x}}{b_{x}{-a}_{x}}b_{y}$$

which is:

$$\left[\begin{array}{cc} b_{y}{-a}_{y} & a_{x}{-b}_{x} \end{array}\right]\left[\begin{array}{c} p_{x}\\ p_{y} \end{array}\right]\leq a_{x}b_{y}{-b}_{x}a_{y}$$

Then, the constraint to be satisfied by CoP at time *k* is:

(22) $$\left[\begin{array}{cc} b_{y}{-a}_{y} & a_{x}{-b}_{x}\\ c_{y}{-b}_{y} & b_{x}{-c}_{x}\\ \vdots & \vdots \\ a_{y}{-f}_{y} & f_{x}{-a}_{x} \end{array}\right]r_{k}^{\mathrm{cop}}\leq \left[\begin{array}{c} a_{x}b_{y}{-a}_{y}{-b}_{x}\\ b_{x}c_{y}{-b}_{y}{-c}_{x}\\ \begin{array}{c} \vdots \\ f_{x}a_{y}{-f}_{y}{-a}_{x} \end{array} \end{array}\right]$$

### 4.3. Cost Function

The cost function is used to ensure that the controller tracks the desired trajectory. This paper adopts the following cost function:

(23) $${J=e}_{k+1}^{T}{\mathrm{Qe}}_{k+1}+{\tilde{r}}_{k}^{\mathrm{cop}}{}^{T}{R\tilde{r}}_{k}^{\mathrm{cop}}$$

where, $e_{k+1}=\xi_{k+1}-\xi_{k+1}^{ref}$, *Q*, and *R* are positive symmetric matrices to correct variables that deviate from the reference trajectory. The cost function can be divided into two parts: the part related to the state space and the part related to the control input. The former is used to reduce the error between the expected and predicted DCM positions, and the latter is used to minimize the CoP rate of change, resulting in a smooth ZMP trajectory.

By merging Formulas (19) and (23) we get the optimization function:

(24) $$\begin{array}{ll} \underset{{\tilde{r}}_{k}^{\mathrm{cop}}}{\min}J & =\underset{{\tilde{r}}_{k}^{\mathrm{cop}}}{\min}{\tilde{r}}_{k}^{\mathrm{cop}}{}^{T}\left[H_{x}^{T}{\mathrm{QH}}_{x}+R\right]{\tilde{r}}_{k}^{\mathrm{cop}}\\ & +2H_{x}^{T}Q\left[P_{x}{\tilde{\xi }}_{x}\right]{\tilde{r}}_{k}^{\mathrm{cop}}+{\left[P_{x}{\tilde{\xi }}_{x}\right]}^{T}Q\left[P_{x}{\tilde{\xi }}_{x}\right] \end{array}$$

In summary, we can find the control input:

(25) $$r_{k}^{\mathrm{cop}}=-{\left[H_{x}^{T}{\mathrm{QH}}_{x}+R\right]}^{-1}H_{x}^{T}{\mathrm{QP}}_{x}{\tilde{\xi }}_{k}$$

### 4.4. Extended Kalman Filter

To effectively deal with the nonlinearities caused by changes in the height of CoM during walking, we use the EKF, which reduces the impact of measurement noise and external disturbances on the system [56,57]. The equations used in the EKF are the same as the ones in [58], where the stages of update and measure are expressed as follows:

Update:

(26) $$\begin{array}{l} {\hat{\xi }}_{k}^{-}{=\hat{\xi }}_{k-1}+w_{k}\\ z_{k}=h\left(y_{k},{\;r}_{k}^{\mathrm{cop}},\;q,\;s\right){+v}_{k}\\ P_{k}^{-}{=A}_{k}P_{k-1}A_{k}^{T}{+W}_{k}Q_{k-1}W_{k} \end{array}$$

Measure:

(27) $$\begin{array}{l} K_{k}{=P}_{k}^{-}H_{k}^{T}{\left(H_{k}P_{k}^{-}H_{k}^{T}{+V}_{k}R_{k}V_{k}^{T}\right)}^{-1}\\ {\hat{\xi }}_{k}{=\hat{\xi }}_{k}^{-}{+K}_{k}\left(y_{k}{-z}_{k}\right)\\ P_{k}=\left({I-K}_{k}H_{k}\right)P_{k}^{-} \end{array}$$

where ${\hat{\xi }}_{k}^{-}$ and ${\hat{\xi }}_{k-1}^{-}$ are the estimated value at now and the last moment. *w_k_* is the system noise. *y_k_* and $r_{k}^{\mathrm{cop}}$ are the output and input respectively, *q* and *s* are the weights, *v_k_* is the measurement noise, and these values determine the output. $P_{k}^{-}$ is the covariance matrix which depends on the system matrix *A_k_*, the covariance matrix of the previous moment *P_k−1_*, the noise covariance matrix *Q_k−1_*, and the system noise matrix *W_k_*. *K_k_* represents the Kalman gain, which depends on *H_k_*, *V_k_*, and $P_{k}^{-}$, where *H_k_* and *V_k_* are matrices of measurement and measuring.

By predicting one step ahead, the EKF updates or revises the forecast using system measurements to estimate the value in the process [59]. A detailed explanation of the above equations can be found in the literature [60]. The matrix *H* determines the magnitude of the Kalman filter gain, which is shown in Formula (28). In summary, the LIPM with finite-sized foot and the optimal algorithm determine the procedure model. In order to obtain the optimal solution and iteratively update the weight matrices, *Q*, *R*, and *H* must be linearized:

(28) $$H=\left[\begin{array}{cc} \frac{\partial h\left(\xi_{1},{\tilde{r}}_{k}^{\mathrm{cop}}\right)}{\partial \xi_{1}} & \frac{\partial h\left(\xi_{1},{\tilde{r}}_{k}^{\mathrm{cop}}\right)}{\partial \xi_{2}}\\ \frac{\partial h\left(\xi_{2},{\tilde{r}}_{k}^{\mathrm{cop}}\right)}{\partial \xi_{1}} & \frac{\partial h\left(\xi_{2},{\tilde{r}}_{k}^{\mathrm{cop}}\right)}{\partial \xi_{2}} \end{array}\right]$$

It is necessary to obtain general expressions for MPC and EKF. Combining Formulas (18) and (25), we can get linear equivalence:

(29) $$\begin{array}{ll} \frac{\partial {\tilde{r}}_{k}^{\mathrm{cop}}}{\partial q}\left({\hat{\xi }}_{1},{\hat{\xi }}_{2}\right)= & -[H_{x}^{T}P_{x}{\hat{\xi }}_{k}\left[H_{x}^{T}{\mathrm{QH}}_{x}+R\right]\\ & -H_{x}^{T}H_{x}\left[H_{x}^{T}P_{x}{\hat{\xi }}_{k}\right]]{\left[H_{x}^{T}{\mathrm{QH}}_{x}+R\right]}^{-2} \end{array}$$

(30) $$\frac{\partial r_{k}^{\mathrm{cop}}}{\partial s}\left({\hat{\xi }}_{1},{\hat{\xi }}_{2}\right)=\left[H_{x}^{T}{\mathrm{QP}}_{x}{\hat{\xi }}_{k}\right]{\left[H_{x}^{T}{\mathrm{QH}}_{x}+R\right]}^{-2}$$

$\xi_{1}$, $\xi_{2}$ are the penalizations *q* and *s*, respectively, which are also used in MPC.

**Figure 5:**
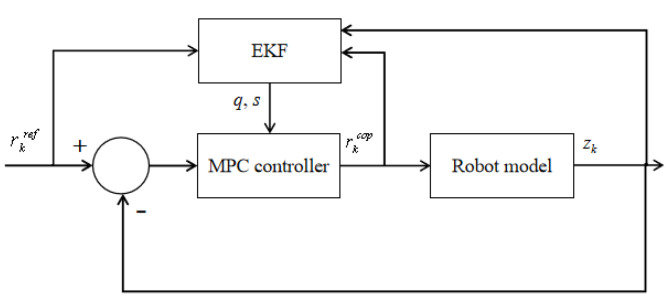
The combination of MPC and EKF.

The state-space variables of the robot model are the DCM in the x and y directions, respectively. We assign the same weight to both directions. In other words, we consider the DCM in the x and y directions to be equally important. The overall control block diagram is shown in Figure 5.

(31) $$\begin{array}{l} Q=q\left[\begin{array}{cc} 1 & 0\\ 0 & 1 \end{array}\right]\\ R=s \end{array}$$

## 5. Experiments

**Figure 6:**
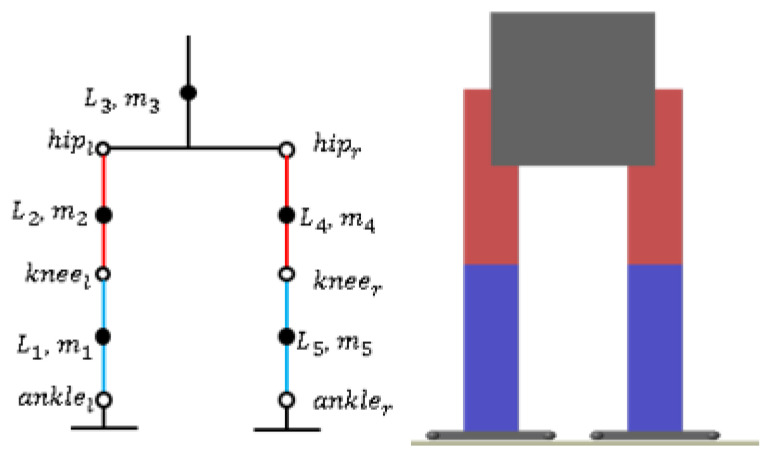
The link structure (**left**) and simulation model (**right**) of the biped robot.

We used matlab2020b as a simulation experiment platform to build a bipedal model. Figure 6 shows the link structure (left) and simulation model (right) of a humanoid robot. Where, *L_i_* and *m_i_* are the length and mass of the links, respectively, i∈{1,2,3,4,5} details are as follows: *L*_1_ = *L*_5_ = 0.38 m, *L*_2_ = *L*_4_ = 0.40 m, *m*_1_ = *m_5_* = 7.6 kg, *m*_2_ = *m*_4_ = 8 kg, and *m*_3_ = 40 kg. The entire model has a total of 12 degrees of freedom, and the number of degrees of freedom of the hip joint, knee joint, and ankle joint are 3, 1, and 2, respectively. The experimental parameters are as follows: the walking period is 1.6 s (0.8 s for one step), the step length is 0.100 m, the step width is 0.140 m, and the maximum height of the swing leg is 0.025 m. To reduce the impact of landing on both feet, the swing-leg trajectory uses cubic polynomial interpolation.

In order to cope with complex environments, humanoid robots should have the ability to walk on uneven ground and cope with external disturbances. Three simulation experiments were designed to demonstrate the effectiveness of the DCM planning and control method.

Simulation 1 The robot walks at a constant speed on a flat surface, and there is no disturbance during the walking.

Simulation 2 The robot walks at a constant speed on flat ground. During the walking, an impact disturbance is applied. The disturbance is applied to the CoM, and it changes the speed and direction of the CoM. In this simulation, the robot stands with a single support on its right leg at 9 s. To obtain the maximum performance of the controller, a disturbance will be applied to the right hip joint at 9 s and last 10 ms, because applying disturbance to the support leg will impact the robot greatly.

Simulation 3 When a robot is walking outdoors, the height of the center of mass of the robot does not remain on a horizontal plane. However, in this case, the calculated DCM trajectory will not guarantee the robustness of the bipedal walking. To ensure that the controller can compensate for the effects of CoM height changes, we built an uneven ground in Simulink and the ground height change was Δh∈[−0.05m,+0.05m]. In this simulation, the robot walks on uneven terrain at a constant speed.

**Figure 7:**
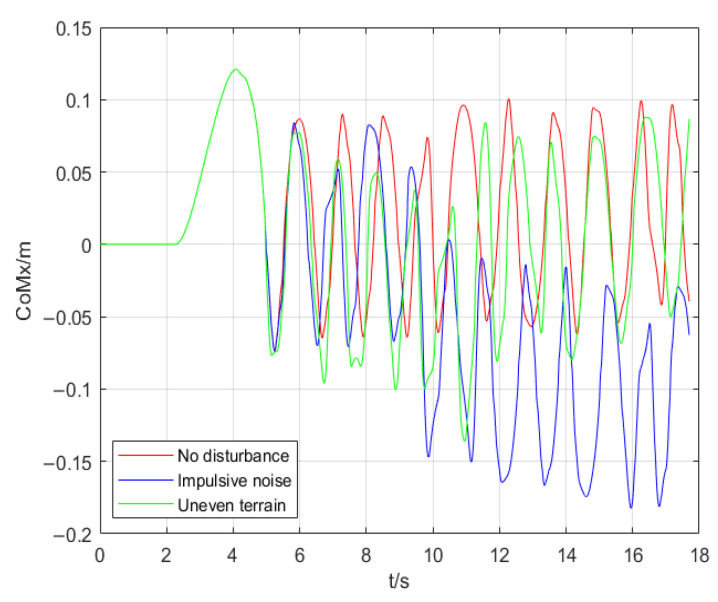
Displacement in the coronal plane of the CoM.

**Figure 8:**
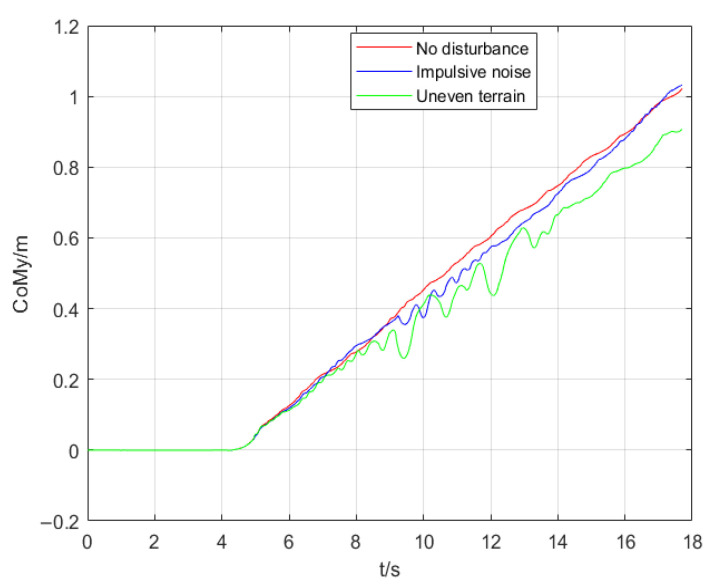
Displacement in the sagittal plane of the CoM.

**Figure 9:**
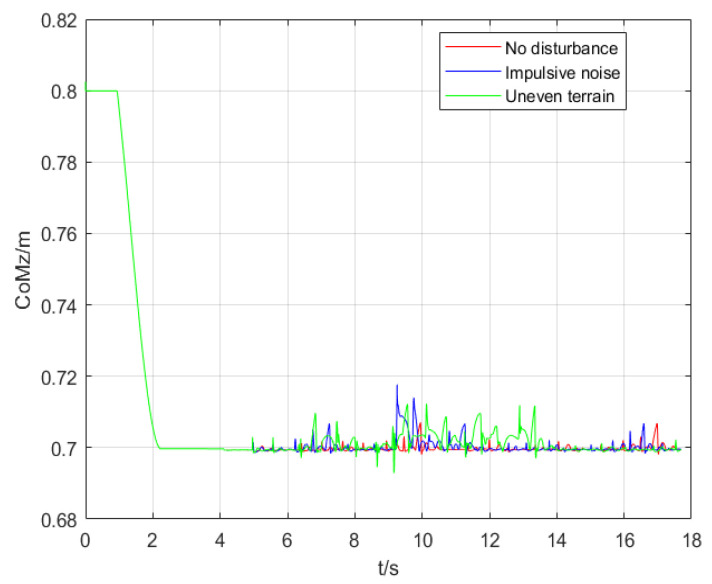
Variation in the height of the CoM.

The results of experiments are compared in Figure 7, Figure 8 and Figure 9. Figure 7 indicates the real CoM position in the coronal plane in three simulations. The CoM position in the sagittal plane is shown in Figure 8. Figure 9 illustrates the height variation of the robot’s CoM in three simulations. The red curve represents the result of simulation 1, the blue curve represents the result of simulation 2, and the green curve describes the result of simulation 3.

Figure 7 shows that the coronal motion of the CoM can be considered as a periodic motion similar to a sinusoid wave when the robot is walking at a constant speed without disturbance. During 0 s to 5 s, the robot is lowering the CoM by bending legs and shifting the CoM to the left leg, so three experiments have the same results. The first two cycles of the robot are walking on flat ground. Simulation 2 (blue curve) shows that when being disturbed by a large shock at 9 s, after 3.5 cycles of correction by the controller, the motion of the coronal plane will balance in a new position and continue to maintain a periodic motion similar to a sinusoid. Thus, the robot takes 7 steps (3.5 cycles) during 9 s to 13 s, one more step than walking on flat ground. Taking one more step in the same amount of time shortens the step length and prevents it from exceeding the maximum limit. Besides this, the robot has one more double-support phase that helps the ZMP stabilize in the center of the support area. Simulation 3 (green curve) shows that when walking on uneven terrain, although the coronal motion of the robot is different from that of undisturbed walking in each cycle, its equilibrium position is always near the equilibrium position of the coronal motion during undisturbed walking. Similar to our description in simulation 2, the controller will adjust the gait of the robot by shortening or lengthening the step time Δ*t* to compensate for the velocity and position errors of CoM. Over all, from Figure 7, we draw the following conclusion: the controller compensates the trajectory error of the CoM by adjusting the step width and increasing the number of DSP.

From Figure 8, it can be seen that when there is no external interference, the sagittal displacement of the CoM is proportional to the time after the robot starts to walk. The red curve shows that when the robot walks on flat ground, it is a uniform linear motion. When the robot is disturbed, there is a small fluctuation in the CoM trajectory, and the fluctuation disappears after the equilibrium is restored. Compared to the desired DCM, the real DCM will produce a larger displacement for the same time interval Δ*t* due to the increased CoM velocity. This will make the step distance of the robot exceed the maximum limit, and even if there is still a margin for the step, a longer step will have a great impact on the next cycle of walking. Thus, the controller shortens the Δ*t* to make the robot’s feet land more frequently. In this way, the robot can adjust the ZMP more quickly, which stabilizes the robot. When walking on uneven terrain (green curve), the sagittal motion of the CoM fluctuates continuously compared with normal walking, but the robot can still walk forward. In this simulation, the velocity of the CoM will increase or decrease. When the velocity of the CoM is increased, the controller will shorten Δ*t*, as we described above. However, the velocity of the CoM may be decreased sometimes when walking on uneven terrain. To solve this problem, the controller lengthens Δ*t*. As shown in Figure 8, during 9.5 s to 10.1s and 12 s to 13 s in simulation 3 (green curve), we can see a significant increase in the step distance of the robot. Overall, from Figure 8, we can see that the controller compensates for the trajectory error of the CoM by adjusting the step length.

Figure 9 indicates that when the robot walks without disturbance, its CoM is always at the same horizontal height. When it is disturbed by impact, its CoM height changes, and gradually returns to the original horizontal height under the action of the controller. The results of the robot walking on uneven terrain show that even if the height of the CoM of the robot changes continuously, it will never fall out of balance.

**Figure 10:**
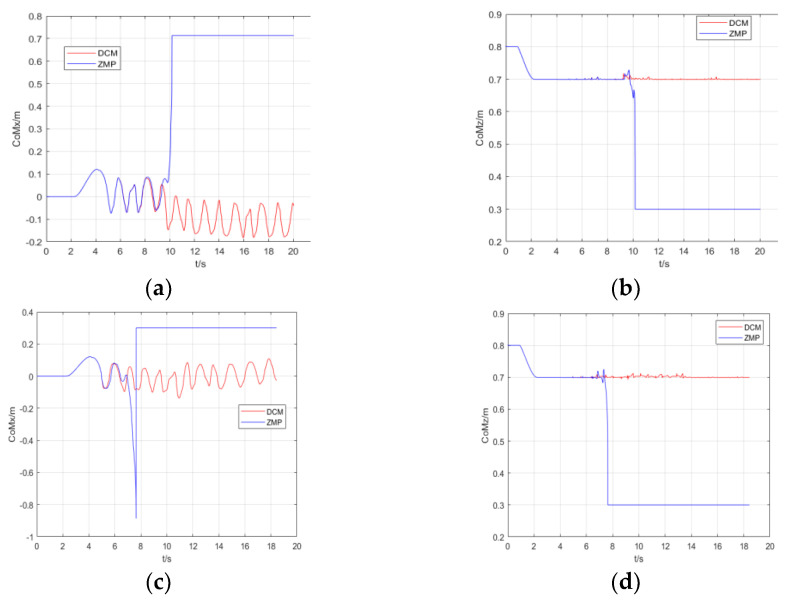
Comparison of ZMP and DCM. (**a**,**b**) Results in simulation 2. (**c**,**d**) Results in simulation 3. (**a**) Displacement in the coronal plane of the CoM. (**b**) Variation in the height of the CoM. (**c**) Displacement in the coronal plane of the CoM. (**d**) Variation in the height of the CoM.

Figure 10 is the comparison of ZMP and DCM in simulation 2 and simulation 3, respectively. Compared to the DCM method, a robot controlled using the ZMP method falls to the left when it suffers an impact disturbance and cannot walk on uneven terrain. The ZMP-based MPC, which uses CoM as the controller input, cannot compensate for displacement and the change of acceleration caused by the external force to the CoM. Walking on uneven terrain causes the height of the robot’s CoM to change constantly; similarly, the acceleration in the z-direction of the CoM can also be difficult to compensate for.

**Figure 11:**
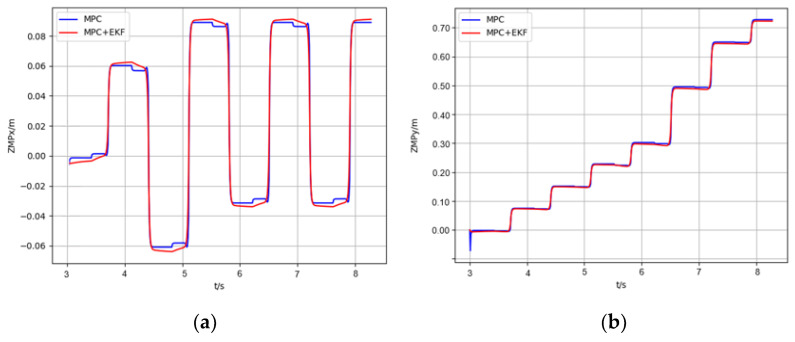
Real ZMP position for MPC and MPC+EKF. (**a**) The real ZMP position in the coronal plane, (**b**) The real ZMP position in the sagittal plane.

Figure 11 shows the real position of ZMP for the MPC and the MPC+EFK method. Figure 11a,b show the real ZMP position in the coronal plane and sagittal plane, respectively. In the coronal plane, the control method we proposed can ensure the ZMP is always positioned on the central axis of the support area and the ZMP trajectory is smooth when it switches between feet. However, the position of the ZMP trajectory is offset from the central axis of the support area and closer to the inside of the foot. Besides, the ZMP trajectory will suddenly change when it switches between two feet. In the sagittal plane, both methods have almost the same result. Overall, the method we proposed generates smoother ZMP trajectories with larger stability margins.

## 6. Conclusions

We propose a robust biped robot gait-generation and control method. We use LIPM with finite-sized feet as the mathematical model of the robot to plan the expected DCM trajectory of the robot in the SSP and DSP. Since the CoM will automatically converge to the DCM, this paper proposes a DCM-based MPC controller to achieve indirect control of the CoM and ZMP by tracking the desired DCM trajectory. The combination of EFK and MPC reduces interference from the outside world. After being disturbed, the robot no longer moves in the desired trajectory. The controller will change the landing point of the actual DCM by optimizing the step time. The change in DCM implies an adjustment in step size and width so that the robot can compensate for CoM velocity and position errors caused by disturbances. Particularly, when the velocity of the CoM is increasing, the controller will shorten the step time to increase the number of DSPs and decrease the step size. It can adjust the ZMP and compensate for the velocity error of the CoM in real time. The experimental results show that the proposed method can effectively deal with the impact disturbance during the walking process of the robot, and at the same time the robot can walk on uneven ground.

In the future, we plan to give different weights to state variables in EKF. This idea is to realize the anthropomorphism of robots. For example, the DCM in the y-direction has a more significant impact on the stability of the robot when a humanoid robot is crossing a bridge or walking on a narrow road. We will let the robot walk on a suspended stone road. It should focus on the lateral external forces when suffering from disturbance. Even if the disturbance cannot be compensated for, it should fall in the forward direction. This can avoid irreparable damage caused by the robot falling from a swell.
